# Non-Alcoholic Fatty Liver Disease and Vitamin D in the UK Biobank: A Two-Sample Bidirectional Mendelian Randomisation Study

**DOI:** 10.3390/nu15061442

**Published:** 2023-03-16

**Authors:** Zixuan Zhang, Kimberley Burrows, Harriett Fuller, Elizabeth K. Speliotes, Kushala W. M. Abeysekera, James L. Thorne, Sarah J. Lewis, Michael A. Zulyniak, J. Bernadette Moore

**Affiliations:** 1 School of Food Science & Nutrition, University of Leeds, Leeds LS2 9JT, UK; 2 Department of Medicine, National University of Singapore, Singapore 117549, Singapore; 3 Population Health Sciences, Bristol Medical School, University of Bristol, Oakfield House, Oakfield Grove, Bristol BS8 2BN, UK; 4 Public Health Science Division, Fred Hutchinson Cancer Center, Seattle, WA 98109, USA; 5 Divisions of Gastroenterology, Computational Medicine and Bioinformatics, Department of Internal Medicine, University of Michigan, Ann Arbor, MI 48109, USA

**Keywords:** non-alcoholic fatty liver disease, NAFLD, vitamin D, 25(OH)D, UK Biobank, Mendelian randomization, MR, GWAS, genetics, epidemiology

## Abstract

Evidence for a role for vitamin D in non-alcoholic fatty liver disease (NAFLD) pathogenesis is conflicting. As Mendelian randomisation (MR) avoids many limitations of conventional observational studies, this two-sample bidirectional MR analysis was conducted to determine the following: (i) whether genetically predicted 25-hydroxyvitamin D [25(OH)D] levels are a risk factor for NAFLD, and (ii) whether genetic risk for NAFLD influences 25(OH)D levels. Single-nucleotide polymorphisms (SNPs) associated with serum 25(OH)D levels were obtained from the European ancestry-derived SUNLIGHT consortium. SNPs associated with NAFLD or NASH (*p*-value < 1 × 10^−5^) were extracted from previous studies and supplemented by genome-wide association studies (GWASs) performed in the UK Biobank. These GWASs were done both without (primary analysis) and with (sensitivity analysis) the population-level exclusion of other liver diseases (e.g., alcoholic liver diseases, toxic liver diseases, viral hepatitis, etc.). Subsequently, MR analyses were performed to obtain effect estimates using inverse variance weighted (IVW) random effect models. Cochran’s Q statistic, MR-Egger regression intercept, MR pleiotropy residual sum and outlier (MR-PRESSO) analyses were used to assess pleiotropy. No causal association of genetically predicted serum 25(OH)D (per standard deviation increase) with risk of NAFLD was identified in either the primary analysis: *n* = 2757 cases, *n* = 460,161 controls, odds ratio (95% confidence interval): 0.95 (0.76, −1.18), *p* = 0.614; or the sensitivity analysis. Reciprocally, no causal association was identified between the genetic risk of NAFLD and serum 25(OH)D levels, OR = 1.00 (0.99, 1.02, *p* = 0.665). In conclusion, this MR analysis found no evidence of an association between serum 25(OH)D levels and NAFLD in a large European cohort.

## 1. Introduction

Non-alcoholic fatty liver disease (NAFLD) is a major cause of chronic liver disease worldwide, affecting approximately 25% of the global population [1]. Defined by the excess accumulation of lipids in the liver, NAFLD encompasses a broad spectrum of liver conditions. These include simple steatosis, also termed non-alcoholic fatty liver (NAFL), non-alcoholic steatohepatitis (NASH), fibrosis, cirrhosis, and hepatocellular carcinoma. The progression of NAFLD depends on complex interactions between genetic risk factors and metabolic comorbidities such as obesity and diabetes [2]. As overnutrition and a sedentary lifestyle are key risk factors driving NAFLD progression, diet and physical activity modification is the cornerstone of NAFLD management guidelines [3]. However, deficiencies in individual dietary nutrients, such as vitamin D, have been implicated in NAFLD pathogenesis and may affect disease development and/or progression through alternative pathways [4]. 

Vitamin D is a misnomer for a family of secosteroid hormones with pleiotropic properties. The most stable circulating form, 25-hydroxyvitamin D, 25(OH)D, has been the most commonly used biomarker of vitamin D status [5], and low serum 25(OH)D levels have been implicated in multiple chronic liver diseases, including NAFLD [6]. In addition, preclinical data suggests that the antiproliferative, anti-inflammatory, antifibrotic, and insulin-sensitising properties of vitamin D may help prevent NAFLD [7,8]. For example, while vitamin D depletion aggravated lobular inflammation and the NAFLD Activity Score (NAS) in high-fat/high-fructose diet-fed rats [9], NAFLD rats treated with vitamin D had reduced liver inflammation and oxidative stress [10]. In humans, observational studies have found an inverse association between vitamin D status and both NAFLD risk, and NAFLD severity [11,12]. Although multiple studies have found lower 25(OH)D concentrations associated with more severe histological features of NAFLD [13,14,15], the results from human intervention trials of vitamin D supplementation in patients with NAFLD have been conflicting [7,16]. 

Mendelian randomisation (MR) is an epidemiological approach that uses genetic variants as instrumental variables (IVs) to determine the causal relationship between an exposure and an outcome in an observational setting [17]. MR avoids many limitations of conventional observational studies, including residual confounding and reverse causation; as the exposures under investigation (i.e., genetic predictors of exposure) are randomised at conception in the absence of confounders [18]. To date, only two MR studies in different ancestries have explored the causal inference between serum 25(OH)D level and NAFLD [19,20]. However, these studies have yielded different results. In a Chinese population, using eight NAFLD-related SNPs and four vitamin D status related-SNPs, Wang and colleagues reported no causal association between genetically predicted 25(OH)D levels and ultrasound-defined NAFLD [19]. In contrast, using seven vitamin D-related SNPs, Yuan and Larsson reported a causal association between higher genetically predicted 25(OH)D levels and lower risk of NAFLD in three populations of European ancestries [20].

As both outcome case definition and choice of instrumental variables can greatly influence MR results, in this study we conducted genome-wide association studies (GWASs) of NAFLD risk in individuals participating in the UK Biobank (UKBB) using a broader case definition than has previously been used. The GWASs were done both without (primary analysis) and with (sensitivity analysis) the population-level exclusion of other liver disease. Then, utilising the genetic instruments extracted from the most extensive meta-analysis GWAS of vitamin D status in a population of European descent conducted to date [21], we conducted a two-sample bidirectional MR analysis to estimate first the effect of genetically predicted 25(OH)D serum levels on risk of NAFLD, and reciprocally to estimate the causal effect of genetic risk for NAFLD on 25(OH)D serum levels.

## 2. Materials and Methods

**Figure 1:**
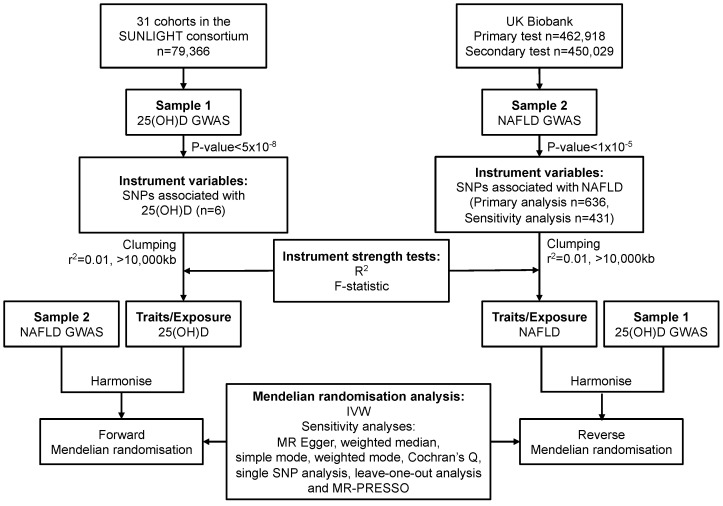
Overview of the two-sample MR study design used to investigate the probability of either a single or bidirectional association between serum 25(OH)D and NAFLD. 25(OH)D, 25-hydroxyvitamin D; GWAS, genome-wide association study; IVW, inverse-variance weighted; MR, Mendelian randomisation; MR-PRESSO, MR pleiotropy residual sum and outlier; NAFLD, non-alcoholic fatty liver disease; SNP, single-nucleotide polymorphism. SNPs were included if identified to be associated with NAFLD in the Speliotes’ GWAS [22] with effects in the same direction with a *p*-value of <1 × 10^−5^ in UKBB or were ‘discovered’ in our GWAS in UKBB with a *p*-value of <5 × 10^−8^.

A summary of the two-sample bidirectional MR design of this study is illustrated in Figure 1.

### 2.1. Study Design and Data Sources

To evaluate the effect of 25(OH)D levels on risk of NAFLD risk (Sample 1, Figure 1) SNPs identified in the Study of Underlying Genetic Determinants of Vitamin D and Highly Related Traits (SUNLIGHT) Consortium [21] that associated with Vitamin D status were used as instrumental variables (IVs) to evaluate the association between genetically predicted serum 25(OH)D levels and NAFLD risk in UK Biobank (UKBB) population cohort. Conversely, in the opposite direction (Sample 2, Figure 1), to evaluate the effect of NAFLD risk on 25(OH)D levels, SNPs associated with NAFLD in previous studies and those identified in UKBB were used as IVs to evaluate their association between NAFLD and 25(OH)D in the SUNLIGHT consortium using summary level data. While the UKBB and SUNLIGHT populations are broadly comparable in terms of their European ancestry, vitamin D status and NAFLD risk, the SUNLIGHT consortium is comprised of 31 cohorts from Europe, Canada and the US.

#### 2.1.1. Data Sources and SNP Selection for Serum 25(OH)D

The summary statistics of the GWAS of serum 25(OH)D concentrations on 2,579,297 SNPs were obtained from the SUNLIGHT Consortium [23]. This meta-analysis collected data from 79,366 individuals from 31 cohorts of European ancestry residing in Europe, Canada and the United States [21]. Specifically, additive genetic models were fitted using linear regression adjusted for month of sample collection, sex, age, BMI and principal components on natural log-transformed 25(OH)D levels. Six SNPs (rs3755967, rs12785878, rs10741657, rs17216707, rs10745742 and rs8018720) with the lowest *p*-value at each locus (all *p*-values < 5 × 10^−8^) were confirmed in two independent in-silico replication cohorts [21]. Therefore, these six SNPs were chosen as IVs for the Sample 1, 25(OH)D exposure on risk of NAFLD MR analysis.

For ease of interpretation of the effect of 25(OH)D exposure on NAFLD, we transformed the SNP effect estimates from the natural log scale to the standard deviation (SD) scale (Supplementary Table S1). Thus, the odds ratios (ORs) of NAFLD were scaled to per SD increase in genetically predicted serum 25(OH)D level. An approximate SD for serum 25(OH)D obtained from the population-based Swedish Mammography Cohort corresponded to 0.33 ln[nmol/L] [24].

#### 2.1.2. Data Sources and SNP Selection for NAFLD

A panel of five SNPs (rs738409, rs2228603, rs4240624, rs780094 and rs12137855) associated with NAFLD in a previously published, relatively small (*n* = 7176), GWAS of European Ancestry from Speliotes and co-workers [22], was a-priori targeted for the analysis. To determine effect sizes in a larger cohort and identify supplemental SNPs associated with NAFLD, a GWAS for NAFLD was undertaken in the UKBB (*n* = 462,918). In our GWAS we also used a more inclusive definition of NAFLD than has previously been used in GWAS. We included SNPs if they had been identified to be associated with NAFLD in the GWAS by Speliotes and had effects in the same direction with a *p*-value of <1 × 10^−5^ in UKBB or were ‘discovered’ in our GWAS in UKBB with a *p*-value of <5 × 10^−8^.

The UKBB is a prospective cohort study that recruited over 500,000 participants aged between 40 and 69 years old between 2006 and 2010 across the UK for genotyping and long term clinical follow up [25]. This rich collection of genomic and deep phenotypical data has facilitated multiple genetic studies in the current literature [26].

In UKBB, the field code 41,270 represents the summary diagnoses of hospital inpatient episodes abstracted from the Hospital Episode Statistic (HES), which are coded according to the International Classification of Disease version 10 (ICD10). The ICD codes K75.8 ‘Other specified inflammatory liver diseases [Nonalcoholic steatohepatitis (NASH)]’, and K76.0 ‘Fatty (change of) liver, not elsewhere classified [Nonalcoholic fatty liver disease (NAFLD), excluded K75.8]’ were used to define NAFLD cases. The number of cases of NAFLD in the UKBB cohort (not subset on genetic data or covariates) from Data-Field 41,270, released in January 2019 was 3044 (Supplementary Table S2). 

We conducted two GWASs of NAFLD cases and controls using UKBB data restricted to European ancestry. In the primary analysis, cases were defined as any participant diagnosed with K75.8 and/or K76.0 at any point during data collection, reflecting the spectrum of NAFLD. Controls were defined as any participant not having a diagnosis of ICD10 codes K75.8 and/or K76.0 at any point during data collection. As it is well known that the genetic risk factors for NAFLD influence multiple liver diseases [27], as a sensitivity analysis, we then filtered the entire UKBB cohort to remove any of the ICD10 codes related to other liver diseases (Supplementary Table S3) and conducted a second GWAS. We then examined the concordance of identified SNPs in the two GWAS. All other aspects of the case definition remained the same. After filtering for participants with genetic data, in total, there were *n* = 2757 cases and *n* = 460,161 controls for the primary analysis, and there were *n* = 1747 cases and *n* = 448,282 controls for the sensitivity analysis.

The UKBB GWASs were conducted using the BOLT-LMM software (version 2.3.2) [28,29] and adjusted for sex and genotype chip. As BOLT-LMM association statistics are on the linear scale, test statistics (β and their corresponding SE) need to be transformed to log ORs and their corresponding 95% confidence intervals (CI) on the liability scale using a Taylor transformation expansion series [29]. SNPs were removed where the minor allele count in cases was <10 [30]. The final number of SNPs included in each GWAS were the following: primary analysis *n* = 11,324,872 (lambdas = 1.05); sensitivity analysis *n* = 10,788,717 (lambdas = 1.00).

Genetic IVs were selected using the TwoSampleMR package [31] in R (v4.1.2, R Develop Core Team, Vienna, Austria) [32] to test the association between geneticlly predicted risk of NAFLD against likelihood of low serum 25(OH)D level (i.e., <50 nmol/mL). Any SNPs previously associated with NAFLD at GWAS significant levels in other studies which were associated with our definition of NAFLD with a *p*-value < 1 × 10^−5^ in UKBB and any new SNPs identified in our study with a genome-wide significance (*p* < 5 × 10^−8^) were selected and pruned by linkage disequilibrium (LD) (r^2^ ≥ 0.01, >10,000 kb) [31,33]. Additionally, the strand direction was inferred using minor allele frequency and palindromic SNPs were removed to prevent strand ambiguity issues [31].

Five SNPs (rs429358, rs3747207, rs9479542, rs10401969 and rs17321515) were associated with NAFLD at *p* < 5 × 10^−8^ in our primary UKBB GWAS. One SNP, rs1260326, related to NAFLD in a previously published study [34], and with a high LD with rs780094 (r^2^ = 0.91) in Speliotes’ study [24], had an effect estimate in the same direction but with a *p* = 1.30 × 10^−7^ in our study. Although, two SNPs (rs429358 and rs3747207) associated with NAFLD were missing in the SUNLIGHT consortium, we were able to identify only one SNP that could be used as a proxy (rs738408, *p* = 9 × 10^−42^) for the SNP (rs3747207) based upon high LD (r^2^ = 0.98). Therefore, five SNP (rs738408, rs9479542, rs1260326, rs10401969, and rs17321515,) were available for the primary test. Similarly, three SNPs (rs3747207, rs4351435 and rs73001065) associated with NAFLD at *p* < 5 × 10^−8^ in our primary GWAS. The rs1260326 with a *p* = 7.99 × 10^−6^ was included. Two SNPs (rs3747207 and rs73001065) were missing in the sensitivity analysis. The SNPs rs738408, and rs10401969 (all *p* < 5×10^−8^) were used as proxies for rs3747207 and rs73001065 (all r^2^ > 0.8), respectively. Thus, four SNPs (rs738408, rs1260326, rs4351435 and rs10149275) were used in the sensitivity test. Notably, three (rs738408, rs1260326, and rs10401969) of the final SNPs were in common between the primary and sensitivity tests.

### 2.2. Statistical Power

In MR, the coefficient of determination (R^2^) can be used as a measure of the proportion of variability in the exposure explained by the IVs [35], while the F-statistic is a measure of the strength of the IV for the exposure of interest. The R^2^ of each IV was estimated and summed to compute the overall R^2^ using the data based on the exposure (Supplementary Figure S1 and Supplementary Tables S4–S6). The F-statistics were assessed for each IV, and the overall F-statistic was the average of all the single study F-statistics (Supplementary Figure S1 and Supplementary Tables S4–S6). In MR analysis, a threshold of F < 10 indicates that the genetic instrument is a weak tool [35,36]. Higher R^2^ and F-statistic values suggest a lower risk of weak instrument bias.

### 2.3. Mendelian Randomisation Analysis

Inverse Variance Weighted (IVW)-random effect was applied as the primary MR analysis method in this study. The IVW-random effect estimates the causal relationship between exposure and outcome by performing a meta-analysis of the ratio of SNP-exposure effects on SNP-outcome effects weighted by the inverse variance of the SNP-exposure effects with heterogeneity adjustment [37,38]. Notably, the IVW method assumes that all the IVs are valid and can return an imprecise estimate if the MR assumptions are not met.

### 2.4. Sensitivity Analysis

Sensitivity analyses using the MR Egger, weighted median, simple mode, and weighted mode methods were also performed [39,40,41]. These methods all make slightly different assumptions; therefore, a consistent effect across multiple methods provides most robust evidence of causal inference [42]. Single SNP analyses were calculated using the Wald ratio to examine the individual effects of SNPs along with the overall results to assess the consistency across SNPs (Supplementary Figure S2) [43]. Additionally, leave-one-out analysis using IVW was conducted by leaving each SNP out of the MR analysis to detect the influential points (Supplementary Figure S3) [44].

Heterogeneity among SNPs included in IVW and MR-Egger analysis was estimated using Cochran’s Q test [45]. The potential for horizontal pleiotropy, where an exposure SNP influences the outcome by mechanisms other than through the exposure, was assessed using the MR Egger regression intercept and MR pleiotropy residual sum and outlier (MR-PRESSO) analyses (Supplementary Table S7) [39,46]. As UKBB was used as a discovery GWAS, and the effect estimates for the SNP-NAFLD associations from this were used in our NAFLD exposure to 25(OH)D MR, our SNP-exposure estimates may be overestimated due to winner’s curse [24]. We therefore conducted a sensitivity IVW analysis using just the three SNPs (rs738409, rs780094 and rs2228603; all *p* < 5×10^−8^) previously identified in Speliotes’ NAFLD GWAS (Supplementary Table S8).

## 3. Results

**Figure 2:**
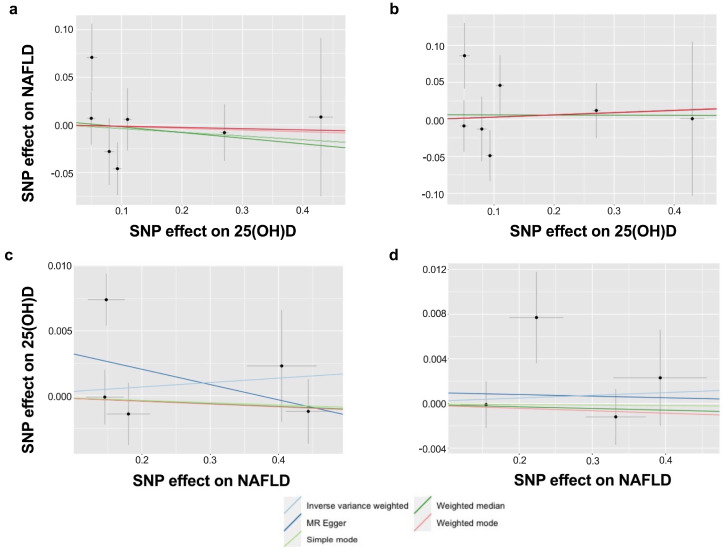
Scatter plots of SNPs associated with 25(OH)D and NAFLD using different MR methods. The genetic associations with circulating 25(OH)D levels against NAFLD risk in the primary analysis (**a**) and sensitivity analysis (**b**). The genetic associations with NAFLD in the primary analysis (**c**) and sensitivity analysis (**d**) against circulating 25(OH)D levels. A cross around each SNP show 95% CI. The slopes of each line represent the causal association for each method. MR, Mendelian randomisation; NAFLD, non-alcoholic fatty liver disease; SNP, single-nucleotide polymorphism.

The IVW-random effect analyses showed no evidence of a causal effect of serum 25(OH)D levels on the odds of NAFLD [OR =0.95 (0.76,1.18); *p* = 0.614, per SD increase; Table 1 and Figure 2], and the OR of the NAFLD sensitivity analysis was 1.04 [(0.79,1.37); *p* = 0.786, per SD increase]. In addition, no causal association was found for the odds of NAFLD on low serum 25(OH)D levels [NAFLD primary: OR = 1.00, 95% CI = 0.99–1.02, *p* = 0.665; NAFLD sensitivity: OR = 1.00 (0.99,1.01), *p* = 0.689].

Sensitivity analyses showed results consistent with the IVW estimates for serum 25(OH)D level on either NAFLD primary or sensitivity test (both Cochran Q-derived P_IVW_ > 0.05, Supplementary Table S7). In addition, no horizontal pleiotropy was detected (P_intercept_  =  0.47 for NAFLD primary test, P_intercept_  =  0.86 for NAFLD sensitivity test). No outliers were identified in the MR-PRESSO analysis. However, the MR IVW analysis of NAFLD in primary test susceptibility in on serum 25(OH) levels showed heterogeneity between instruments (*I*^2^  = 63.42% for IVW, *I*^2^  = 50.65% for MR Egger; both Cochran Q-derived P_IVW_ <  0.05), but without evidence of horizontal pleiotropy (MR-Egger, P_intercept_  =  0.47). Additionally, no outlier was identified by the MR-PRESSO in this analysis. The sensitivity IVW analysis using three SNPs previously identified in the Speliotes’ GWAS [22] indicated that no causal association was found for the risk of NAFLD (primary test) on odds of low serum 25(OH)D levels [OR =1.01 (0.99,1.02); *p* = 0.563].

## 4. Discussion

In this comprehensive, two sample, bidirectional MR analysis of vitamin D status and NAFLD, we find no evidence to support either a single or bi-directional causal association between serum 25(OH)D levels and risk of NAFLD in a large cohort of European ancestry.

Despite a large body of preclinical and observational data for a relationship between vitamin D status and risk and severity of NAFLD, robust evidence of causality from clinical intervention trials remains lacking [7,16]. While a systematic review and meta-analysis of sixteen randomized controlled trials in patients with NAFLD concluded that vitamin D supplementation could beneficially affect multiple anthropometric and biochemical indices (e.g., body weight and ALT) [47], a Cochrane review that more broadly focused on chronic liver disease in adults reported that vitamin D supplementation had no effect on liver function or steatosis in patients with NAFLD, but it cautioned that the evidence base for this (11 trials) was extremely weak [48]. Our own systematic review of vitamin D intervention trials in NAFLD found that only 6 of 13 identified studies that used ultrasound or transient elastography measurements for NAFLD diagnosis reported significant improvement in the grade of steatosis or fibrosis in adults at the postinterventional point, and study quality was variable [49].

Although MR avoids many limitations of conventional epidemiological studies, to date, only two studies have used MR to assess the causal relationship between 25(OH)D and NAFLD [19,20]. Done in different populations (one Chinese, one European), these have also drawn contradictory conclusions [19,20]. While a one-sample bidirectional MR analysis in a Chinese population did not support a causal relationship between vitamin D and NAFLD [19]; a two-sample bidirectional MR meta-analysis of three European cohorts found evidence of a causal effect between higher serum 25(OH)D and a decreased risk of NAFLD [20]. In contrast, our study found no evidence to support a relationship between serum 25(OH)D levels and risk of NAFLD in a large cohort of European ancestry.

While multiple methodological differences between studies are likely to partially explain the contradictory results; in particular, how NAFLD is defined will impact case prevalence estimates and with it, MR results. For instance, NAFLD prevalence may be underreported in population-based healthcare databases using ICD codes due to delays in data updating, compared to clinical data updated directly from the electronic patient medical records [50]. This likely contributes to the difference between our results (using UK Biobank ICD codes) and those of the meta-analysis by Yuan and Larsson [20], which included a cohort of clinically defined NAFLD cases from specialized European liver centres [51], in addition to two healthcare databases (UKBB [52] and FinnGen Consortium GWAS [53]) that used only the ICD10 K76.0 criteria for diagnosis [53]. However, the most likely cause of discordance was our examination of NASH and NAFLD as outcomes, which positions our work as an investigation on the wider spectrum of liver conditions associated with NAFLD and its progression.

Our study design had several strengths. First, as NAFLD is an umbrella term for a wide range of liver conditions [4], we chose to define cases inclusively and used both ICD10 K75.8 (NASH) and K76.0 (NAFLD) for case definition for this study. This resulted in 2757 cases in the primary GWAS analysis and for the vitamin D to NAFLD MR analysis, comparable to the 2652 used in the overall meta-analysis of Yuan and Larsson [20]. Second, we conducted two NAFLD GWASs, one with, and one without, “other liver diseases” to evaluate pleiotropy between “other liver diseases” and NAFLD. Of the identified SNPs, three overlapped between the two GWAS using different definitions of NAFLD, and included well known NAFLD risk associated loci, such as the patatin-like phospholipase domain-containing protein 3 (*PNPLA3*)), transmembrane 6 superfamily 2 (*TM6SF2*), and glucokinase regulatory (*GCKR*) genes. Third, the SUNLIGHT Consortium and the UKBB data were generated from two independent European populations, which avoided potential false-positive findings from participant overlap. Lastly, the vitamin D IVs used in the current study were chosen from the largest vitamin D GWAS with biological plausibility at the gene level for most of the variants with respect to circulating vitamin D [54] and replication in other studies [55,56].

Nonetheless, this study had some limitations. First, the data used in the current two-sample MR were summary-level genetic data from two large GWASs that used different covariable adjustments (e.g., the vitamin D GWAS adjusted for month of sample collection, while UKBB did not). Covariable-adjusted summary associations may introduce bias to the analyses, including residual confounding between covariable and outcome [57]. Second, NAFLD encompasses a broad spectrum of liver conditions, and the progression of NAFLD has considerable heterogeneity among its subtypes [58]. However, due to limitations of the HES data in the UKBB, and due to low power, sub-analyses examining vitamin D effects on different stages of NAFLD (NAFL/NASH/fibrosis/cirrhosis) could not be performed. Third, our analyses of the effect of NAFLD on vitamin D are potentially susceptible to the winner’s curse [59], because we conducted a GWAS of NAFLD in UKBB and used the effect estimates for the instruments identified to be associated with NAFLD in this cohort in our Mendelian randomization analysis. However, we do not believe that this biased our results substantially because when we restricted our analysis to those SNPs that were previously identified to associated with NAFLD by Speilotes et al. (sensitivity analysis, Supplementary Table S8), we obtained similar results. Additional limitations come from the known selection bias in the UKBB cohort and possible underestimation of NAFLD prevalence from ICD code diagnosis [50,60]. Lastly, these analyses were done in populations of European ancestry so may not be translatable to other populations. Indeed, it may be that European populations are more sensitive to environmental conditions rather than genetics for lifetime vitamin D exposure so the effect may be minimized or masked in this group.

In conclusion, causal effects between either vitamin D status and the risk of NAFLD, or the genetic risk of NAFLD on vitamin D status, were not found in this MR analysis. Although larger cohorts and future meta-analyses across different populations may shed light, improving the accuracy of population-based data on the prevalence of NAFLD will be imperative to epidemiologic strategies. These data will also be influenced by the outcomes of the ongoing, global, Delphi-based, NAFLD Nomenclature Consensus Process [61].

## Figures and Tables

**Table 1:** Results of two-sample bidirectional MR analysis of the causal effects between 25(OH)D serum levels and NAFLD.

Outcome	Method	Number of SNP	OR (95% CI)	*p*-Value
25(OH)D vs. NAFLD				
Primary ^1^	IVW-random effects	6	0.95 (0.76–1.18)	0.641
	MR Egger		0.91 (0.59–1.39)	0.684
	Weighted median		0.97 (0.80–1.18)	0.761
	Simple mode		0.99 (0.69–1.41)	0.951
	Weighted mode		0.97 (0.78–1.20)	0.790
Sensitivity ^1^	IVW-random effects	6	1.04 (0.79–1.37)	0.786
	MR Egger		1.00 (0.59–1.71)	0.990
	Weighted median		1.03 (0.80–1.32)	0.824
	Simple mode		0.88 (0.55–1.41)	0.612
	Weighted mode		1.04 (0.79–1.35)	0.801
NAFLD vs. 25(OH)D				
Primary ^2^	IVW-random effects	5	1.00 (0.99–1.02)	0.665
	MR Egger		0.99 (0.96–1.02)	0.523
	Weighted median		1.00 (0.99–1.01)	0.670
	Simple mode		1.00 (0.99–1.01)	0.789
	Weighted mode		1.00 (0.99–1.01)	0.701
Sensitivity ^2^	IVW-random effects	4	1.00 (0.99–1.01)	0.689
	MR Egger		1.00 (0.96–1.04)	0.951
	Weighted median		1.00 (0.99–1.01)	0.817
	Simple mode		1.00 (0.98–1.02)	0.960
	Weighted mode		1.00 (0.99–1.01)	0.763

25(OH)D, 25-hydroxyvitamin D; CI, confidence interval; IVW, Inverse variance weighted; M, model; MR, Mendelian randomisation; NAFLD, non-alcoholic fatty liver disease; OR, odds ratio; SNP, single-nucleotide polymorphism. ^1^ These analyses are SD-scale ORs; ^2^ these analyses are log-scale ORs.

## Data Availability

The original contributions presented in the study are included in the article/Supplementary Materials, further inquiries can be directed to the corresponding author.

## Supplementary Materials

The following supporting information can be downloaded at: https://www.mdpi.com/article/10.3390/nu15061442/s1, Figure S1: The equations of R^2^ and F-statistic calculation; Figure S2: Forest plots of results of single-SNP Mendelian randomisation; Figure S3: Forest plots of results of leave-one-out Mendelian randomisation; Table S1: Transformation from log-scale to SD-scale on the genetic instruments for the serum 25(OH)D to NAFLD analysis; Table S2: Cases of NAFLD in the entire UK Biobank cohort; Table S3: International Classification of Disease version 10 (ICD-10) codes and case numbers excluded prior to second (sensitivity analysis) GWAS; Table S4: Summary of genetic variants used to estimate the effect of serum 25(OH)D levels on NAFLD; Table S5: Summary of genetic variants used to estimate the effect of NAFLD (primary test) on serum 25(OH)D levels; Table S6: Summary of genetic variants used to estimate the effect of NAFLD (secondary test) on serum 25(OH)D levels; Table S7: Sensitivity analyses; Table S8: Sensitivity IVW analysis using a panel of SNPs previously identified in the Speliotes GWAS [22] on estimating the effect of NAFLD (primary test) on serum 25(OH)D levels; Table S9: Case definition and numbers of NAFLD GWASs for the causal effects of serum 25(OH)D levels on the risk of NAFLD. Ref. [62] is cited in the Supplementary Materials.
