# Supplementary material for: Non-Alcoholic Fatty Liver Disease and Vitamin D in the UK Biobank: A Two-Sample Bidirectional Mendelian Randomisation Study
Source: Nutrients. 2023 Mar 16;15(6):1442. doi: 10.3390/nu15061442 (PMC10058870; doi:10.3390/nu15061442)
Supplement: Supplementary file 1 [file nutrients-15-01442-s001.zip › nutrients-2244026-supplementary.pdf]

SUPPLEMENTARY FIGURES AND TABLES

Non-alcoholic Fatty Liver Disease and Vitamin D in the UK Biobank: A Two-sample Bidirectional Mendelian Randomisation Study

Zixuan Zhang, Kimberley Burrows, Harriett Fuller, Elizabeth K Speliotes, Kushala W M Abeysekera, James L Throne, Sarah J Lewis, Michael A Zulyniak* and J Bernadette Moore*

*Correspondence: M.A.Zulyniak@leeds.ac.uk or J.B.Moore@leeds.ac.uk

Table of Contents

Supplementary Table S1 Transformation of the genetic instruments from log-scale to SD-scale on for the serum 25(OH)D versus NAFLD analysis 1

Supplementary Table S2 Cases of NAFLD in the UK Biobank[1] 2

Supplementary Table S3 International Classification of Disease version 10 (ICD-10) codes and case numbers excluded prior to second (sensitivity analysis) GWAS 3

Supplementary Figure S1 The equations of R^2 and F-statistic calculation. (a) Equation of R^2 calculation; (b) Equation of F-statistic calculation. 4

Supplementary Table S4 Summary of genetic variants used to estimate the effect of serum 25(OH)D levels on risk of NAFLD 5

Supplementary Table S5 Summary of genetic variants used to estimate the effect of NAFLD (primary analysis) on serum 25(OH)D levels 6

Supplementary Table S6 Summary of genetic variants used to estimate the effect of NAFLD (sensitivity analysis) on serum 25(OH)D levels 7

Supplementary Figure S2 Forest plots of results of single-SNP Mendelian randomization. 8

Supplementary Figure S3 Forest plots of results of leave-one-out Mendelian randomisation ... 9

Supplementary Table S7 Sensitivity analyses 10

Supplementary Table S8 Sensitivity IVW analysis using a panel of SNPs previously identified for estimating the effect of NAFLD (primary test) on serum 25(OH)D levels 11

Supplementary Table S9 Case definition and numbers of NAFLD GWASs for the causal effects of serum 25(OH)D levels on the risk of NAFLD 12

Supplementary Table S1 Transformation of the genetic instruments from log-scale to SD-scale on for the serum 25(OH)D versus NAFLD analysis

SNP	Gene	EA/NEA	EAF	Sample size	Log odds scale		SD scale[1]	
					Beta (β)	SE	Beta (β)	SE
rs3755967	GC	T/C	0.28	78232	-0.089	0.002	-0.270	0.007
rs12785878	NADSYN1	T/G	0.75	78328	0.036	0.002	0.110	0.007
rs10741657	CYP2R1	A/G	0.40	78328	0.031	0.002	0.093	0.007
rs17216707	CYP24A1	T/C	0.79	71484	0.026	0.003	0.080	0.008
rs10745742	AMDHD1	T/C	0.40	69167	0.017	0.002	0.050	0.007
rs8018720	SEC23A1	C/G	0.82	68134	-0.017	0.003	-0.051	0.009

[1]The beta and SE of the SD scale were transformed from the log odds scale by dividing the beta and SE of log odds by 0.33 [23].

Abbreviations: AMDHD1, amidohydrolase domain containing 1; CYP2R1, cytochrome P450 2R1; CYP24A1, cytochrome P450 24A1; EA, effect allele; NEA, non-effect allele; EAF, effect allele frequency; GC, vitamin D-binding protein; NADSYN1, nicotinamide adenine dinucleotide synthetase 1; SE, standard error; SEC23A1, SEC homolog A1; SNP, single-nucleotide polymorphism.

Supplementary Table S2 Cases of NAFLD in the UK Biobank[1]

	K76.0 No	K76.0 Yes	Total
**K75.8 No**	0	2696	2696
**K75.8 Yes**	186	162	348
**Total**	186	2858	3044

[1]The number of cases not subset on genetic data or co-variates from Data-Field 41270, released in January 2019.

K75.8, ‘Other specified inflammatory liver diseases [Nonalcoholic steatohepatitis (NASH)]’;

K76.0, ‘Fatty (change of) liver, not elsewhere classified [Nonalcoholic fatty liver disease (NAFLD), excluded K75.8]’.

Supplementary Table S3 International Classification of Disease version 10 (ICD-10) codes and case numbers excluded prior to second (sensitivity analysis) GWAS

Exclusion ICD codes or self-reported	Subcategory code	UKBB Cases
K70 Alcoholic liver disease	K70.0, K70.1, K70.2, K70.3, K70.4, K70.9	1921
K71 Toxic liver disease	K71.0, K71.1, K71.2, K71.3, K71.4, K71.5, K71.6, K71.7, K71.8, K71.9	105
K72 Hepatic failure, not elsewhere classified	K72.0, K72.1, K72.9	617
K73 Chronic hepatitis, not elsewhere classified	K73.0, K73.1, K73.2, K73.8, K73.9	226
K74 Fibrosis and cirrhosis of liver	K74.0, K74.1, K74.2, K74.3, K74.4, K74.5, K74.6	1565
K75 Other inflammatory liver diseases	K75.0, K75.1, K75.2, K75.3, K75.4, K75.9	810
K76 Other diseases of liver	K76.1, K76.2, K76.3, K76.4, K76.5, K76.6, K76.7, K76.8, K76.9	3573
K77 Liver disorders in diseases classified elsewhere	K77.0, K77.8	25
B15 Acute hepatitis A	B15.0, B15.9	97
B16 Acute hepatitis B	B16.0, B16.1, B16.2, B16.9	136
B17 Other acute viral hepatitis	B17.0, B17.1, B17.2, B17.8, B17.9	215
B18 Chronic viral hepatitis	B18.0, B18.1, B18.2, B18.8, B18.9	701
B19 Unspecified viral hepatitis	B19.0, B19.9	47
B94.2 Sequelae of other and unspecified infectious and parasitic diseases	B94.2	2
C22 Malignant neoplasm of liver and intrahepatic bile ducts	C22.0	241
E83 Disorders of mineral metabolism	E83.0	8
E83 Disorders of mineral metabolism	E83.1	973
I85 Oesophageal varices	I85.0	138
I98 Other disorders of circulatory system in diseases classified elsewhere	I98.2	394
G93 Other disorders of brain	G93.7	1
R17 Unspecified jaundice	R17	1294
R18 Ascites	R18	2475
R94 Abnormal results of function studies	R94.5	5505
T86 Failure and rejection of transplanted organs and tissues	T86.4	31
Z94 Transplanted organ and tissue status	Z94.4	146
		Total 21,246

Supplementary Figure S1

(a)

R^2 equation for each SNP:

$$R^2 = \frac{-2 \times (\beta^2) \times EAF \times (1\text{-}EAF)}{2 \times (\beta^2) \times EAF \times (1\text{-}EAF) + (SE^2) \times (2 \times n) \times EAF \times (1\text{-}EAF)}$$

Abbreviations: β, beta of exposure; EAF effect allele frequency; n, size of exposure GWAS; SE, standard error.

(b)

F-statistic equation for each SNP:

$$F = \frac{(R^2) \times (n - 2)}{1 - R^2}$$

Note: n=size of exposure GWAS.

Supplementary Figure S1 The equations of R^2 and F-statistic calculation. (a) Equation of R^2 calculation; (b) Equation of F-statistic calculation [62] .

Supplementary Table S4 Summary of genetic variants used to estimate the effect of serum 25(OH)D levels on risk of NAFLD

SNP	Gene	EA/ NEA	EAF	Sample size	Association with vitamin D					Association with NAFLD Primary analysis			Association with NAFLD Sensitivity analysis		
					Beta	SE	P-val	R^2	F-Statistic	Beta	SE	P-val	Beta	SE	P-val
rs3755967	GC	T/C	0.28	78232	-0.270	0.007	**2.23×10^{-300}**	1.87%	1491.06	8.12×10^{-3}	2.97×10^{-2}	0.78	-1.20×10^{-2}	3.73×10^{-2}	0.75
rs12785878	NADSYN1	T/G	0.75	78328	0.110	0.007	**3.80×10^{-62}**	0.34%	269.55	5.74×10^{-3}	3.28×10^{-2}	0.86	4.59×10^{-2}	4.11×10^{-2}	0.26
rs10741657	CYP2R1	A/G	0.40	78328	0.093	0.007	**2.05×10^{-46}**	0.28%	193.92	-4.60×10^{-2}	2.75×10^{-2}	0.095	-4.91×10^{-2}	3.45×10^{-2}	0.16
rs17216707	CYP24A1	T/C	0.79	71484	0.080	0.008	**8.14×10^{-23}**	0.13%	94.47	-2.79×10^{-2}	3.50×10^{-2}	0.43	-1.30×10^{-2}	4.40×10^{-2}	0.77
rs10745742	AMDHD1	T/C	0.40	69167	0.050	0.007	**1.88×10^{-14}**	0.08%	55.69	6.88×10^{-3}	2.79×10^{-2}	0.81	-8.88×10^{-3}	3.50×10^{-2}	0.8
rs8018720	SEC23A1	C/G	0.82	68134	-0.051	0.009	**4.72×10^{-9}**	0.05%	33.46	7.08×10^{-2}	3.54×10^{-2}	**0.045**	-8.61×10^{-2}	4.44×10^{-2}	0.053
								2.76% (Sum)	356.36 (Mean)						

Abbreviations: AMDHD1, amidohydrolase domain containing 1; CYP2R1, cytochrome P450 2R1; CYP24A1, cytochrome P450 24A1; EA, effect allele; NEA, non-effect allele; EAF, effect allele frequency; GC, vitamin D-binding protein; NADSYN1, nicotinamide adenine dinucleotide synthetase 1; SE, standard error; SEC23A1, SEC homolog A1; SNP, single-nucleotide polymorphism.

Supplementary Table S5 Summary of genetic variants used to estimate the effect of NAFLD (primary analysis) on serum 25(OH)D levels

SNP	Gene	EA/ NEA	EAF	Sample size	Association with NAFLD					Association with vitamin D		
					Beta	SE	P-val	R^2	F-statistics	Beta	SE	P-val
rs738408	PNPLA3	C/T	0.78	462918	-0.443	0.033	**8.96×10^{-42}**	0.0396%	183.36	1.20×10^{-3}	2.50×10^{-3}	0.65
rs1260326	GCKR	T/C	0.40	462918	0.146	0.028	**1.30×10^{-7}**	0.0060%	27.86	-1.00×10^{-3}	2.10×10^{-3}	0.97
rs9479542	RP11-15G8.1	A/G	0.76	462918	0.180	0.032	**1.55×10^{-8}**	0.0069%	31.99	-1.40×10^{-3}	2.40×10^{-3}	0.58
rs10401969	SUGP1	T/C	0.92	462918	-0.405	0.051	**2.27×10^{-15}**	0.0136%	62.81	-2.30×10^{-3}	4.30×10^{-3}	0.59
rs17321515	NA	A/G	0.53	462918	0.148	0.027	**4.63×10^{-8}**	0.0065%	29.87	7.40×10^{-3}	2.00×10^{-3}	**1.77×10^{-4}**
								0.07% (Sum)	67.18 (Mean)			

Abbreviations: EA, effect allele; NEA, non-effect allele; EAF, effect allele frequency; GCKR, glucokinase regulator; PNPLA3, patatin-like phospholipase domain-containing protein 3; SE, standard error; SUGP1, SURP and G-patch domain-containing protein 1; Notably, the primary test was a GWAS of NAFLD cases and controls without excluding other liver diseases.

Supplementary Table S6 Summary of genetic variants used to estimate the effect of NAFLD (sensitivity analysis) on serum 25(OH)D levels

SNP	Gene	EA/ NEA	EAF	Sample size	Association with NAFLD					Association with vitamin D		
					Beta	SE	P-val	R^2	F-statistics	Beta	SE	P-val
rs738408	PNPLA3	C/T	0.78	450029	-0.332	0.041	**7.22×10^{-16}**	0.0145%	65.07	1.20×10^{-3}	2.50×10^{-3}	0.65
rs4351435	RP11-136O12.2	G/A	0.30	450029	0.224	0.037	**1.40×10^{-9}**	0.0081%	36.66	7.70×10^{-3}	4.10×10^{-3}	0.06
rs1260326	GCKR	T/C	0.40	450029	0.155	0.035	**7.99×10^{-6}**	0.0044%	19.94	-1.00×10^{-4}	2.10×10^{-3}	0.97
rs10401969	SUGP1	T/C	0.92	450029	-0.393	0.064	**9.16×10^{-10}**	0.0083%	37.50	-2.30×10^{-3}	4.30×10^{-3}	0.59
								0.0354% (Sum)	39.79 (Mean)			

Abbreviations: EA, effect allele; EAF, effect allele frequency; GCKR, glucokinase regulator; NEA, non-effect allele; PNPLA3, patatin-like phospholipase domain-containing protein 3; SE, standard error; SNP, single-nucleotide polymorphism; SUGP1, SURP and G-patch domain-containing protein 1.

Supplementary Figure S2

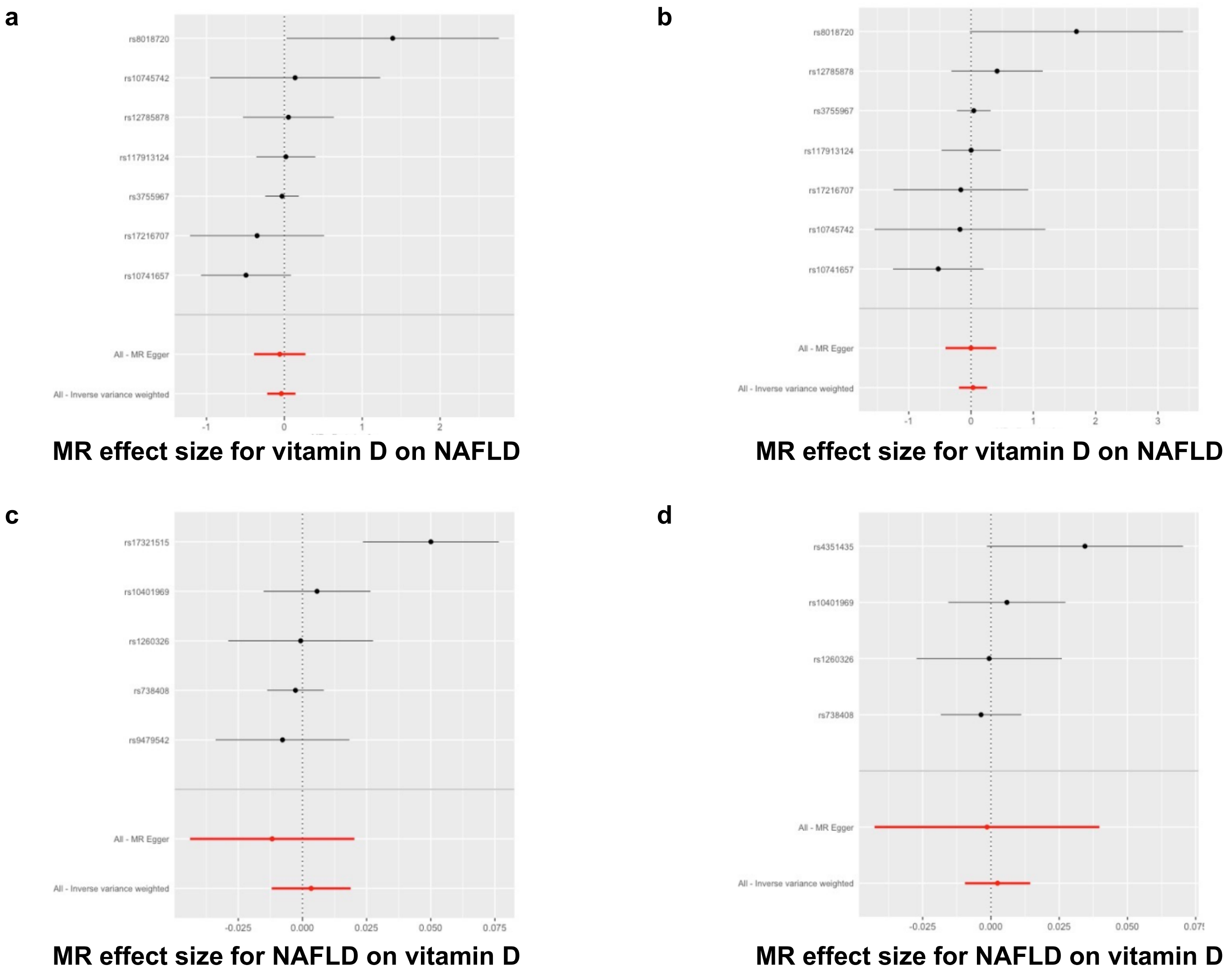

Supplementary Figure S2 Forest plots of results of single-SNP Mendelian randomization. Forest plots of the six serum 25(OH)D SNPs associated with NAFLD risk in primary test (a) and secondary (sensitivity) test (b). Forest plots of the 10 NAFLD SNPs in the primary test (c) and the six NAFLD SNPs in the sensitivity test (d) associated with circulating 25(OH)D level. MR, Mendelian randomisation; NAFLD, non-alcoholic fatty liver disease; SNP, single-nucleotide polymorphism.

Supplementary Figure S3

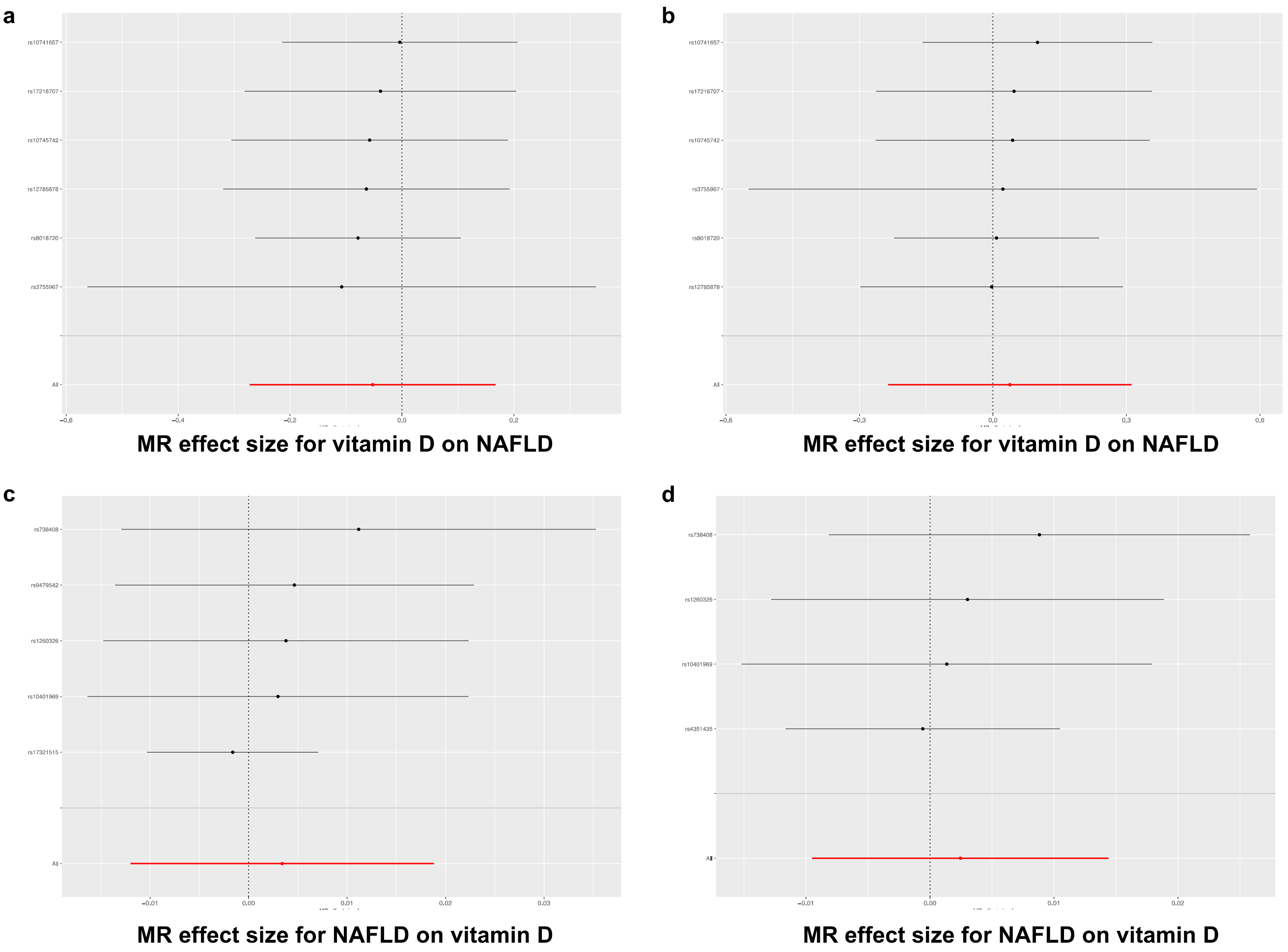

Supplementary Figure S3 Forest plots of results of leave-one-out Mendelian randomisation. Forest plots of the leave-one-out analysis (using IVW) excluding each of the six serum 25(OH)D SNPs associated with NAFLD risk in primary test (a) and secondary (sensitivity) test (b). Forest plots excluding each of 5 NAFLD SNPs in the primary test (c) and each of the four NAFLD SNPs in the sensitivity test (d) associated with circulating 25(OH)D level. MR, Mendelian randomisation; NAFLD, non-alcoholic fatty liver disease; SNP, single-nucleotide polymorphism.

Supplementary Table S7 Sensitivity analyses

Exposure/Outcome	Methods	Test of heterogeneity			Test of pleiotropy		
		**Q**	**P-value**	**I^2**	**Intercept**	**SE**	**P-value**
**Serum 25(OH)D level/ NAFLD (primary test)**	IVW	7.280	0.20	31.32%	-	-	-
	MR Egger	7.175	0.13	30.31%	0.0072	0.0297	0.82
	MR-PRESSO: Global test P-value=0.43, no outlier identified.						
**Serum 25(OH)D level / NAFLD (sensitivity test)**	IVW	7.172	0.21	30.28%	-	-	-
	MR Egger	7.130	0.13	29.88%	0.0057	0.0371	0.89
	MR-PRESSO: Global test P-value=0.45, no outlier identified.						
**NAFLD (primary test)/ Serum 25(OH)D level**	IVW	13.896	**0.008**	64.02%	-	-	-
	MR Egger	10.132	**0.017**	50.65%	0.0044	0.0042	0.37
	MR-PRESSO: Global test P-value=0.09, no outlier identified.						
**NAFLD (sensitivity test)/ Serum 25(OH)D level**	IVW	3.840	0.28	21.88%	-	-	-
	MR Egger	3.767	0.15	20.35%	0.0011	0.0055	0.86
	MR-PRESSO: Global test P-value=0.40, no outlier identified.						

Abbreviations: IVW, Inverse variance weighted; NAFLD, non-alcoholic fatty liver disease; MR, Mendelian randomisation; MR-PRESSO, MR Pleiotropy RESidual Sum and Outlier; SE, standard error.

Supplementary Table S8 Sensitivity IVW analysis using a panel of SNPs previously identified for estimating the effect of NAFLD (primary test) on serum 25(OH)D levels

Exposure	Outcome	Method	Number of SNP	OR[1] (95% CI)	P-value
rs738409, rs780094 and rs2228603[2]	Vitamin D	IVW-random effects	3	1.01 (0.99-1.02)	0.563

[1] Log-scale

[2] These three SNPs from Speliotes' NAFLD GWAS [24] were in high LD with rs738408, rs1260326 and rs10401969, respectively, in the current UKBB NAFLD GWAS.

Abbreviations: IVW, Inverse variance weighted; NAFLD, non-alcoholic fatty liver disease; OR, odds ratio; SNP, single-nucleotide polymorphism.

Supplementary Table S9 Case definition and numbers of NAFLD GWASs for the causal effects of serum 25(OH)D levels on the risk of NAFLD

NAFLD GWAS	Population	Definition of cases	Cases (n=)	Controls (n=)	Data source
The Neale lab [52]	UKBB[1]	ICD10 K76.0	275	360,919	The Neale lab (http://www.nealelab.is/uk-biobank)
FinnGen [53]	Finnish Biobank	ICD10 K76.0	894	217,898	FinnGen consortium (http://www.finngen.fi/fi)
Anstee *et al.* [51]	Clinically defined NAFLD[2]	Abnormal biochemical tests and US bright liver, associated with MS features; or abnormal biochemical tests and macroscopic appearances of steatosis during bariatric surgery	1,483	17,781	PubMed ID 32298765
Zhang et al. Primary UKBB	UKBB[3]	ICD10 K75.8 and K76.0	2,757	460,161	See section 2.1.2 Data Sources and SNPs Selection for NAFLD
Zhang et al. Sensitivity UKBB	UKBB[4]	ICD10 K75.8 and K76.0	1,747	448,282	See section 2.1.2 Data Sources and SNPs Selection for NAFLD

[1]UKBB data released in August 2018

[2]From leading European tertiary liver centres

[3]UKBB data release in January 2019

[4]UKBB data release in January 2019 excluding other liver diseases (Supplementary Table S3)

Abbreviations: GWAS, genome-wide association study; ICD10, International Classification of Disease version 10; NAFLD, non-alcoholic fatty liver disease; UKBB, UK Biobank
